# Chronic Kidney Allograft Disease: New Concepts and Opportunities

**DOI:** 10.3389/fmed.2021.660334

**Published:** 2021-07-14

**Authors:** Sergi Codina, Anna Manonelles, Maria Tormo, Anna Sola, Josep M. Cruzado

**Affiliations:** ^1^Department of Nephrology, Hospital Universitari Bellvitge, Barcelona, Spain; ^2^Institut d'Investigació Biomèdica de Bellvitge (IDIBELL), Hospitalet de Llobregat, Barcelona, Spain; ^3^Department of Clinical Sciences, University of Barcelona, Barcelona, Spain

**Keywords:** review, transplantation, kidney disease, graft survival, regeneration

## Abstract

Chronic kidney disease (CKD) is increasing in most countries and kidney transplantation is the best option for those patients requiring renal replacement therapy. Therefore, there is a significant number of patients living with a functioning kidney allograft. However, progressive kidney allograft functional deterioration remains unchanged despite of major advances in the field. After the first post-transplant year, it has been estimated that this chronic allograft damage may cause a 5% graft loss per year. Most studies focused on mechanisms of kidney graft damage, especially on ischemia-reperfusion injury, alloimmunity, nephrotoxicity, infection and disease recurrence. Thus, therapeutic interventions focus on those modifiable factors associated with chronic kidney allograft disease (CKaD). There are strategies to reduce ischemia-reperfusion injury, to improve the immunologic risk stratification and monitoring, to reduce calcineurin-inhibitor exposure and to identify recurrence of primary renal disease early. On the other hand, control of risk factors for chronic disease progression are particularly relevant as kidney transplantation is inherently associated with renal mass reduction. However, despite progress in pathophysiology and interventions, clinical advances in terms of long-term kidney allograft survival have been subtle. New approaches are needed and probably a holistic view can help. Chronic kidney allograft deterioration is probably the consequence of damage from various etiologies but can be attenuated by kidney repair mechanisms. Thus, besides immunological and other mechanisms of damage, the intrinsic repair kidney graft capacity should be considered to generate new hypothesis and potential therapeutic targets. In this review, the critical risk factors that define CKaD will be discussed but also how the renal mechanisms of regeneration could contribute to a change chronic kidney allograft disease paradigm.

## Introduction

CKD is and will be one of the biggest threats for health systems worldwide due to its prevalence and cost, especially when referring to kidney replacement therapy (1). Nowadays, kidney transplantation is the treatment of choice since it has shown its superiority at improving survival and quality of life and reducing costs and comorbidity (2). The incidence of this technique has increased in recent years reaching a median rate of 33 pmp in Europe (3). Therefore, there is a significant number of patients living with a functioning kidney allograft, particularly in high-income countries. On the other side, progressive allograft function loss has become one of the most important causes of KRT requirement. Looking back, kidney allograft survival has undoubtedly improved. While in the 90's the median survival for kidney grafts was nearly 6.6 years, the current expected lifespan is about 8.8 years (4). However, this gain in allografts expected lifetime is mostly due to the improvement in short time graft survival (4). After the first post-transplant year, it has been estimated that chronic allograft damage may cause a 5% graft loss per year and this rate has seen very little decline in recent years (5). As a result, while short-term graft survival is relatively ensured, the tendency to improve long-term graft survival seems to have slowed down if we compare recent cohorts (3, 5).

From a clinical point of view, there is a wide spectrum of etiologies that explain graft damage (6). Currently, therapeutic interventions target those modifiable factors associated with kidney allograft attrition: reduce ischemia-reperfusion injury, minimize calcineurin-inhibitor exposure, control of classical risk factors or early identification of recurrence of primary renal disease. In addition, a lot of efforts are focused on improving immunologic risk stratification and on the development of new therapeutic strategies against kidney's rejection (7). Separately, all these etiologies can contribute in a major or minor form to the loss of renal mass that will end in chronic graft disease progression (8, 9).

Histologically, chronic graft damage means almost always fibrosis. Fibrosis develops in many patients during the first year after transplantation, especially in the first 3 months (10). Because most of the knowledge we have in this field is based on protocol biopsies, it is difficult to precisely titter the prevalence of fibrosis in allograft, but it varies from 17 to 66% according to the series (11, 12). However, fibrosis will continue to grow in the following years, especially in those patients with more fibrosis in former biopsies (12–14). This progression happens despite the absence or minimization of risk factors (13). Currently, alloimmunological damage is thought to be the main cause of kidney allograft fibrosis (15).

In short, a set of insults from different etiologies are capable of harming the kidney graft and altogether contribute to one common outcome. In this review, we will use the terminology chronic kidney allograft disease (CKaD) to refer to that global damage. We prefer the term CKaD in spite of other formulas (i.e., chronic allograft nephropathy) to emphasize CKaD as a global entity that summarizes the sum of all the damages of each etiology, not only immunological. In addition, we like the similarity with the term CKD as it helps to understand CKaD as a chronic, progressive, multifactorial disease, and above all, as a disease that requires a specific approach with nephroprotective strategies. In fact, despite the progress made in each area, clinical advances in terms of long-term kidney graft survival have been subtle (3) so new approaches are needed and the concept CKaD aims to provide a more holistic point of view. It is important to understand that chronic allograft deterioration is the consequence not only of the damage from various etiologies but also of the imbalance of kidney's own repair mechanisms (16). Based on recent investigations, it can be hypothesized that besides immunological and other mechanisms of damage, the graft's intrinsic capacity to repair could attenuate kidney damage and should be considered in new therapeutic approaches. In this review, the critical risk factors that define CKaD will be discussed but also how the knowledge on kidney mechanisms of repair could contribute to change the CKaD paradigm.

## The Harms

There are several mechanisms of kidney allograft damage and the most relevant will be described in this part (Table 1). Individually, all of them cause nephron loss on a solitary kidney and potentially activate the key mechanism of kidney disease progression by producing glomerular hypertension and hyperfiltration. Renin angiotensin blockade and SGTL-2 inhibitors could alleviate this common mechanism of CKD progression in native and transplanted kidneys. However, this topic is beyond the scope of this revision.

**Table 1:** Harms from different etiology contribute to CKaD.

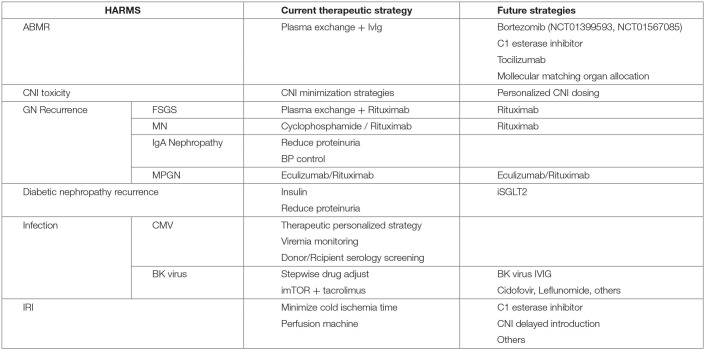

### Anti-body Mediated Rejection (ABMR)

Antibody-mediated rejection (ABMR) has been recognized as a major cause of organ-transplant failure during the past two decades (17). Nowadays, it is widely accepted that ABMR is the major risk factor for the development of CKaD and it can explain more than half of graft losses (18). Although there is some lack of knowledge about its natural history yet, understanding about ABMR has drastically increased in last years.

ABMR clinical impact increases poor graft and patient survival outcomes (19). In a large cohort of 885 kidney transplant recipients who underwent biopsies for graft dysfunction, patients with ABMR morphology showed an 8-year graft survival of 53% in C4d-positive and 66% in C4d-negative. This rate was significantly lower than the 81% seen in patients without any rejection features (20). To notice, the presence of complement-fixing antibodies, the extent of graft dysfunction at baseline and the presence of chronic lesions (capillary multilayering, arterial intimal fibrosis, glomerular basement membrane double contours) are associated with the poorest outcomes (21, 22). The main risk factor known for ABMR development is the number of HLA mismatches and poor adherence to medications.

Briefly, its pathogenesis is explained by the existence or *de novo* apparition of donor-specific antibodies (DSA). DSAs recognize some molecules expressed on the recipient's endothelium and this initiates an inflammatory cascade that leads to tissue damage. Among others, the classical pathway of the complement has been identified as an important way for antibody mediated damage (23). This simplistic explanation was the basis for the first definition of ABMR in the Banff classification from 1997 and allowed to stablish three standardized criteria for its diagnosis: First, identification of DSA. Second, evidence of endothelial damage. Third, proof of complement mediated damage through Cd4 staining.

Though, ABMR pathogenesis is more complex and further information has forced to readapt its definition until the actual one (24). Due to its predominant role in ABMR, DSA against HLA antigens are commonly screened in clinical practice, especially if rejection is suspected. However, DSA are not always targeted to HLA antigens but to other endothelial antigens (23). Thus, the current definition of ABMR accepts the diagnostic even without an identified DSA as long as evidence of antibody mediated damage exists (24). In addition, although complement activation is the most deleterious mechanism of allograft injury (25), antibody mediated harm also occurs through other pathways (26). Consequently, ABMR can also be diagnosed in C4d negative cases if DSA are present or in chronic ABMR.

No therapeutic strategy has received FDA approval for the treatment of antibody-mediated rejection. Based on ABMR pathogenesis, plasma exchange plus IVIG to remove circulant DSAs is considered the current standard of care despite low quality evidence (27, 28). Two trials were designed to test the effect of Rituximab plus plasmapheresis and/or IVIG on ABMR in order to decrease the production of DSA. Both trials ended with underpowered results showing no benefit on short graft survival nor on GFR loss prevention (29, 30). Furthermore, targeting plasmatic cells has not shown the desired effect. The BORTEJECT trial was based on the anti-proteosome drug Bortezomib. No differences in GFR attrition were observed but more gastrointestinal and hematological adverse events were observed in the Bortezomib group (31). Two more randomized clinical trials are currently active and will test Bortezomib (ClinicalTrials.gov numbers, NCT01399593 and NCT01567085). The aforementioned impact of the complement cascade in ABMR has been another therapeutic approach. To that end, the anti-C5 antibody Eculizumab, has been used both for ABMR treatment and for ABMR prevention in desensitization protocols. Despite some trends suggesting marginal beneficial effects (32), at this point of time Eculizumab has also failed to prevent graft damage secondary to ABMR (33, 34). Instead of targeting the end of the cascade, another valid strategy could be point to the top through C1 esterase inhibitors. In a phase II study with plasma derived C1 esterase inhibitor, Montgomery et al. (35) showed a secure profile of the treatment and described that no transplant glomerulopathy was found in the treatment group whereas transplant glomerulopathy were present in 3 of 7 patients in the control group. Another therapeutic approach with promising result is the use of the anti-IL-6 antibody Tocilizumab, which in a French cohort of KTR with refractory cABMR showed a graft survival of 80% at 6 years, with a reduction in glomerulitis, peritubular capilaritis and C4d deposition in allograft biopsies after treatment (36). RCTs are currently going on to confirm these therapeutic options. Other new strategies tested in ongoing registered trials are corticotropin or double filtration plasmapheresis (13). However, it should be pointed out that there is a surprising discrepancy between the theoretic high ABMR prevalence and the lack of power of interventional clinical trials on chronic ABMR due to the lack of patients fulfilling inclusion criteria.

Due to the lack of a solid therapeutic approach, prevention of ABMR occurrence may be crucial. Nowadays, to talk about ABMR prevention means improvement of compatibility. Scientific literature is full of evidence showing the correlation with more HLA matches and better graft outcomes (37). However, HLA incompatible transplantations cannot be avoided in current health systems. The study of these HLA miss-matched transplants has identified that some of these miss-matches confers more risk (Unacceptable miss-match) while others are better tolerated (acceptable mismatches) (38, 39). Differences in permissibility between HLA-mismatched combinations may be explained by a different impact of amino-acid polymorphisms on peptide-binding features. Some tools have been developed to calculate this binding-site or epitope miss-match to further compatibility stratification. In a Dutch multi-center study, 2,918 donor–recipient couples were retrospectively stratified using one of these tools (Predicted Indirectly ReCognizable HLA Epitopes presented by recipient HLA class II: PIRCHE-II) (40). For these donors–recipients couples, PIRCHE-II numbers were related to graft survival in univariate and multivariable analyses. Recently, DQ-antibody verified eplet miss-match has showed to be associated with an increased risk of dnDSA formation, kidney rejection, decline of graft function and graft failure (41). However, no study has reported benefits of these tools in a decision-making prospective protocol.

In short, ABMR is the main cause of long-term graft loss and so far, we have failed to dramatically improve its outcomes. In the absence of effective therapeutic approaches, prevention of ABMR occurrence plays a key role. Probably, molecular matching techniques will be included in organ allocation schemes in the next years. Early treatment and avoiding fibrosis could be essential to prevent long-term allograft attrition.

### Calcineurin Inhibitor (CNI) Toxicity

The importance of CNI toxicity on CKaD has drastically decreased over the decades (42). Putting on some perspective, CNI toxicity leading to chronic damage was pointed out after Myers et al. (43) published a case-control study of cardiac transplant recipients under treatment with Cyclosporine for at least 12 months. In this study, 2 patients developed ESKD and the median eGFR of patients treated with CNI presented an important attrition in comparison with controls. Since then, CNI related toxicity leading to ESKD has been observed in other solid organ transplantation cases. Nevertheless, more than three decades have passed and there have been some changes in CNI paradigm: first, the better knowledge of antibody mediated damage and the development of new diagnostic techniques has brought out the important role of alloimmunity in CKaD (see above). Second, CNI levels have been taken to the minimum necessary to ensure safety for both patient and graft survival. Third, Tacrolimus more than Cyclosporine is the current CNI of choice in many cases reducing associated adverse events (44, 45). After all these changes, CNI toxicity has been displaced as a cause of CKaD by some authors. In 2012 Sellarés et al. (18) tried to find a diagnostic explanation beyond “chronic graft nephropathy” for every graft loss in a cohort of 315 patients. In that cohort, no graft failure was attributed to CNI toxicity.

How can CNI toxicity as cause of CKaD have gone from all to nothing? Probably, there are a set of characteristics that make CNI toxicity something like Santa: “you can only see if you believe in it”:

a) On one hand, existence of CNI toxicity should be beyond doubt. Its damage mechanisms have been characterized, with an acute reversible damage related to an imbalance between vasodilators and vasoconstrictors and tubular vacuolization; and a chronic damage secondary to reduced glomerular blood flow with ischemic-reperfusion mediated injury (46). In the other hand, when large sparing CNI clinical trials were performed, differences in graft function seemed to be more related to the introduction of IL-2 antibody and MMF optimization than to CNI dosage (7, 47, 48).
b) CNI toxicity is dose-dependent (49, 50). However, the range of tacrolimus levels in those studies were between 5 and 25 ng/ml and the actual recommended target levels are much narrower, conferring less significant differences (51).
c) Histologically, chronic damage due to CNI toxicity shows arteriolar hyalinosis, interstitial fibrosis, tubular atrophy, juxtaglomerular apparatus hyperplasia, glomerular capsular fibrosis and global glomerulosclerosis. Unfortunately, none of these findings are pathognomonic, have not any specific marker and its presence can be explained by other common graft comorbidities.
d) Finally, some factors which are not measured in clinical practice could explain important differences in CNI susceptibility. For example, local concentration of Cyclosporine in kidney is associated with more susceptibility to toxicity and chronic damage (52). However, local concentration does not correlate with systemic blood levels and has a great interindividual variation due to genetic differences and drug interactions. Similarly, while some authors have described the presence of DNA SNPs that confers and increased risk of CNI toxicity (53), personalized CNI dosing according to genetic SNPs exists but is not globally extended.

In conclusion, CNI toxicity is an exclusion diagnosis without any specific marker. From a pragmatic point of view, while in other solid organ transplants chronic kidney damage could reasonably be attributed to CNI toxicity (54); in kidney transplantation is hard to find a situation in which graft loss occurs without any other comorbidity that could contribute to chronic renal damage. Thus, isolated chronic CNI toxicity diagnostic is always controversial and should be performed with caution. However, beyond a categorical diagnostic, CNI effects on renal function and chronic damage through fibrosis are probably always present, contributing to global graft attrition and therefore to CKaD.

### Glomerulonephritis (GN) Recurrence

GN recurrence accounts for a large amount of kidney graft recipients evolving to end stage kidney disease. Its exact proportion varies from 3 to 18% of graft losses according to the series (55, 56) but probably, GN recurrence is under diagnosed in transplant recipients (57). There are four main entities that explain the majority of cases of GN recurrence: focal segmental glomerulosclerosis (FSGS), membranous nephropathy (MN), membranoproliferative GN (MPGN), and IgA nephropathy (IgAN). In 2017, Cosio and Cattran (58) published an outstanding review on this topic. So, in this paper we will focused on the key points and actualize the issue with current evidence (Table 2).

**Table 2:** Recurrence profile of the main Glomerulonephritis (GN).

**GN**		**Recurrence**	**Risk Factors**	**Graft loss**	**Treatment**
FSGS		30 %	- Aggressive primary disease - Recurrence in former graft - Non-genetic (except NPH2 mutation)	30%	PlasmapheresisRituximab
MN		30-75%	- Anti-PLA2R + at diagnostic - Elevated Anti-PlA2R titter at transplant or after - HLA-D and PLA2R specific mutations	50% of recurrence when graft loss occurs	Rituximab or Cyclophosphamide
IgA Nephropathy		30% Late recurrence	- Not well-defined - Steroid withdrawal	10%	Avoid steroid withdrawal
MPGN	Polyclonal Ig	30%	- Low C3/C4 blood levels	10%	Rituximab
	Monoclonal Ig	70%		50%	
	C3GN	70%	- alterations in the regulation of the alternative pathway of the complement cascade		Eculizumab (?)
	DDD	80–90%		25%	

*FSGS, Focal segmental glomerulosclerosis; MN, Membranous nephropathy; MPGN, Membranoproliferative glomerulonephritis; C3GN, C3 glomerulonephritis; DDD, Dense deposit disease. The symbol (?) pretend to express that evidence is scarce*.

### Recurrent FSGS

FSGS is a common cause of recurrence reaching 30% in 3 years. Of these, nearly 30% will advance to graft loss (58). FSGS recurrence occurs early after transplantation. Genetic forms of FSGS are less probable to appear in the allograft except for podocin mutations (NPHS2) (59). Clinical predictors of GN recurrence are those signs of disease severity in the primary episode: evolution to dialysis in <3 years after the diagnosis and high levels of proteinuria after transplantation. Classically, young age is associated with more recurrence though some new evidence contradicts this idea (60). Unfortunately, these factors are imprecise and the most reliable predictor of GN recurrence is the recurrence itself in a former graft.

Although the pathogenesis of primary FSGS remains unknown, the clinical profile after transplantation hardly suggests the presence of a circulating factor (61). In a blind attempt to remove this factor, plasmapheresis is commonly used to treat GN recurrence. The TANGO project aims to create an international collaborative data sharing network about GN recurrence in order to generate stronger evidence on the field (62). As a result of this project, Uffing et al. (60) showed that in current clinical practice the therapeutic response after plasmapheresis was nearly 50%. The second most common pharmacological approach seen in this study was the use of Rituximab. Whether the beneficial effect of Rituximab is through targeting B lymphocyte or directly to podocyte SMPDL-3b protein is currently under discussion (63). Curiously, in the TANGO study there is an almost despicable use of Cyclosporine despite previous data suggested a potentially beneficial effect combined with Plasmapheresis (64).

### Recurrent MN

Globally, recurrence of MN is about 50%. However, its range varies from 30 to 75%. This variation is in close relationship with anti-PLA2R titter profile. Thus, primary MN anti-PLA2R mediated has more risk of recurrence than no antibody mediated disease (58, 65, 66). In addition, the presence of persistent elevated titters of antibodies after transplantation carries a higher risk of recurrence. Also, genetic factors have been associated to MN risk of recurrence. For example, the presence of SNP mutations on HLA-D and PLA2R loci confer more risk of recurrence when presented by the donor (67). Most recipients with recurrent MN are under CNI treatment, an effective therapy in native kidneys. Another line of treatment is the use of alkylating agents such as cyclophosphamide, but it has a high risk of medullar toxicity specially if treatment with MMF is not stopped. Current evidence suggest that the use of Rituximab is an effective and safe strategy for these patients, with the additional advantage that do not precise the adjustment of the other immunosuppressors (66, 68) though further studies are needed.

### Recurrent IgA Nephropathy

Prevalence of recurrent IgA Nephropathy is 30% according to clinical reports (69). However, two major considerations must be made: first, unlike other primary diseases, IgA recurrence occurs later, even 10 years after transplantation, so some series may underestimate real prevalence due to short follow-up. Second, the histological immunofluorescence pattern characteristic of the disease appears in a very large proportion of patients much before clinical manifestation (70). Though, it should be noticed that some apparently normal donors (living or deceased) may have latent IgA deposits in the kidney (71). Currently, there is no way to know which latent IgA will evolve to clinical recurrence or will otherwise disappear. No robust evidence about other risk factors for IgA recurrence exists, although some authors suggest that primary disease's activity (presence of crescents, rapid evolution to ESKD) could be associated with higher rate of recurrence (72). IgA nephropathy impact on graft survival is mild and survival graft curves just differ from other entities beyond 10 years post transplantation (69). Histological Oxford classification could have prognostic value for allograft failure (73). No specific treatment is recommended for IgA recurrence. According to KDIGO guidelines, treatment should aim to reduce proteinuria, to optimize blood pressure and to reduce inflammation (74). Steroid withdrawal is associated with major incidence of recurrence and poorer graft outcomes (75). Studies from Japan reported favorable outcomes after tonsillectomy, but these results need to be confirmed (76).

### Recurrent MPGN

Each type and subtype of MPGN has its particular evolution after transplantation (58). In general, MPGN due to Immunoglobulin (Ig) deposition has a lower risk of recurrence (30–70%) specially if the Ig are polyclonal. Instead, recurrence of MPGN due to complement deposition rises to 70–90%. Regarding MPGN with Ig deposition, polyclonal Ig MPGN usually appears after some years and presents a slow course (77). Instead, recurrence by monoclonal Ig occurs more often, earlier and is associated with a more aggressive course that often leads to graft failure (77). On the other hand, both DDD and G3GN are associated with a very high risk of recurrence. In addition, C3GN is associated with poor graft survival (78). Treatment of Ig deposition associated MPGN targets Ig production through Rituximab. Though the absence of CT some series has shown promising response rates (79, 80). As C3GN and DDD patients have presented alterations in alternative pathway of the complement cascade, there are several reports on the use of monoclonal antibodies that inhibit activation of the C5 component of complement (eculizumab) with distinct results (81, 82) but so far, no RCT has been done.

### Diabetic Nephropathy and Other Classical CKD Risk Factors

Diabetic nephropathy is not only the main cause of ESKD but is also associated with greater morbidity and mortality when it occurs in the kidney graft (83, 84). Post-transplant diabetic nephropathy (PTDN) shares pathophysiological and histological characteristics with primary diabetic nephropathy (85). However, the associated complications seem to develop at an accelerated rate (86). Older age, and obesity have shown to be the main risk factors for the development of PTDN (87). In addition, early low-grade proteinuria (<0.3 g/day) and hypertension (especially systolic blood pressure and elevated pulse pressure) have also been described as risk factors (88). Strategies with reduction or avoidance of steroids tend to decrease the incidence of PTDN (89). CNI are also associated with a high risk of developing PTDN. Among them, Cyclosporine seems to be less hyperglycemic according to the Diabetes Incidence after Renal Transplantation trial (DIRECT) (90). However, Tacrolimus continues to have a safer cardiovascular profile due superior lipid, blood pressure and kidney function effects (91).

The treatment of PTDN does not differ from native diabetic nephropathy. Screening for PTDM should be performed after starting treatment with glucocorticoids, sirolimus, or CNI (74). The choice of oral medication vs. insulin treatment must be done under the exact same rationale than in diabetic nephropathy. To mention, there's little evidence that basal insulin initiation to avoid hyperglycemic status immediately post transplantation can prevent further presentation of PTDN (92). In patients who develop PTDN with overt micro and macroalbuminuria, use of angiotensin inhibitors and statins are strongly recommended. This recommendation is an extrapolation from the effects observed in general population. However, in transplant population with proteinuria of any cause, treatment with angiotensin inhibitor has not demonstrated any benefit in long-term graft survival (93). Lately, iSGLT2 have shown remarkable results in decreasing cardiovascular risk and increasing survival both in general and in chronic kidney disease population (94–96). Given the enthusiasm that they have caused, it was predictable that some papers would appear defending that its use in kidney transplant recipients is safe and effective (97–100). Certainly, randomized trials will attempt this issue soon.

Beyond diabetic nephropathy, there are other risk factors of kidney disease progression but its impact in CKaD are not clearly defined. Briefly, hypertension is a common risk factor of renal disease progression, especially if it is associated with proteinuria. The use of RAAS blockade is one of the main strategies to reduce CKD progression. Hypothetically, the same mechanisms of damage occur on the kidney graft and may lead to CKaD but when it has been studied, results are not clear. While some retrospective cohorts associated the use of ACEI/ARB to better graft and patient survival (101, 102), prospective trials have not confirmed these association with graft survival (93, 103). The effect of obesity in graft outcomes is also controversial. Lafranca et al. (104) recently reviewed its effect, showing better outcomes for graft survival in patients with low BMI (<30). However, patient survival expressed in hazard ratios was in significant favor of high BMI recipients. Hyperlipidemia has also shown important outcomes related to patient survival but not related with CKAD. Though the anti-inflammatory effects of statins, associated with the inhibition of HMG CoA, have reported some good results in other solid organ transplantation (105) its use in kidney transplantation hasn't reported strong evidence in decreasing CKaD (106).

### Infections

Infections are one of the most common complications after kidney transplantation due to immunosuppression (107). Besides of its impact in mortality, infections are a well-known risk factor for graft loss (108). Two different scenarios are especially important in relation to CKaD: first, the majority of infections are bacterial urinary tract infections (UTI). They occur more in the elderly due to immunosenescence, frailty, functional impairment and multiple comorbidities (109, 110). Female gender and obesity are also risk factors for the development of urinary infections. They usually occur within the first year after transplantation (111, 112). Despite screening of asymptomatic bacteriuria being common in Kidney Transplant Units (113), its treatment hasn't proved to reduce the incidence of acute pyelonephritis (114). Some series have demonstrated the association between the presence of UTI in the first year after transplant and poorer graft outcomes (108, 115). To explain this, we could extrapolate the effect of acute pyelonephritis on renal scarring and nephron mass loss observed in children (116). However, the association between UTI and loss of kidney functions has been observed also in patients with one or a few episodes of infection in which major renal mass loss is not expected (108). Another explanation is that infections and its clinical context could lead to an immunological imbalance triggering a rejection that would be the culprit for the dysfunction. Interestingly, in one of the few papers that has evaluated this issue, acute pyelonephritis was not independently associated with long term graft survival (117). Altogether, acute pyelonephritis is the most common complication in graft survival and has severe consequences on both patient's and graft's survival.

Second, viral infections play a relevant role in the development of CKaD. Cytomegalovirus (CMV) is the most infectious pathogen in KT recipients and it has been associated with both poor patient and graft survivals as it has been associated with cardiovascular mortality and an increased risk for acute rejection (118). Luckily, the better knowledge and prevention strategies have led to a drastic reduction of the prevalence of CMV infection from 40–100% to 0–37% (118). On the contrary, human polyomavirus has significantly increased its prevalence and it is associated with an important number of graft losses. BK virus nephropathy (BKVN) is an entity that occurs in up to 10% of renal transplant recipients and can result in graft loss in up to 50% of those affected (119). BK virus is a human polyomavirus of high prevalence and low morbidity with an estimated prevalence in adults of 80– 90% (120). After infection, BK virus may establish itself in a state of non-replicative asymptomatic infection in the renal epithelial and urothelial cells (121). In the host, BK virus can reactive itself in context of both immunosuppression and cellular injury (“two-hit hypothesis”). Three stages of the disease have been described: BKVN starts with viral cytopathic effects (stage A), then leads to an inflammation phase (stage B) and finally tubular atrophy and interstitial fibrosis (stage C) (122). The last stage would explain irreversible graft damage and then CKAD. The lack of strategies to prevent or treat BKVN explain the ominous prognosis of the entity with respect to graft survival. Since treatment options are limited and have poor results, strategies to prevent BKVN are crucial. In kidney transplant units, it is common to perform BK virus surveillance by monitoring BKV viremia post-transplant at different time points (123). This monitoring combined with stepwise drug adjustment is the strategy most commonly used and has become the gold standard. We must assume that lowering immunosuppression implies an increased risk of T-cell-mediated rejection (TCMR) and antibody-mediated rejection (ABMR) (124–126). Based on observations from DIRECT (92) and TRANSFORM (127) trials, treatment with ciclosporin and mTOR seemed to be better than Tacrolimus, although the combination of everolimus and tacrolimus was associated with lower incidence of BKVN than MMF with tacrolimus (128, 129). Some other interventions have been attempted with IVIG, cidofovir or Leflunomide among others. Some papers show 90% clearance of BK viremia and sustained graft function after 12 months with IVIG (130–132). Cidofovir has shown to stop polyomavirus replications *in vitro*, as well as good results on achieving BK clearance in a total of 11 cases in the literature (133–135). Conversion from MPA/MMF to Leflunomide has shown therapeutic response in some studies (136) but not in others (137).

### Cold Ischemia Time and Ischemia Reperfusion Injury (IRI)

After surgical removal of the organs for transplantation, kidneys are stored in a cold solution to preserve their viability. Cold ischemia time (CIT) is defined as the time that passes from surgical graft removal until the organ is warmed by recipient's blood supply after artery unclamping. CIT is a well-known risk factor for the development of delayed graft function (DGF) (138, 139) and acute rejection (AR) (140). This association implies both poorest graft and patient survival for those transplants with more CIT. Importantly, CIT itself does not seem to determine a decrease in long term graft survival (140–142).

Despite there are many definitions, DGF is the termed used in literature for those grafts that need at least one dialysis session in the first week after kidney transplantation. A longer CIT expanded criteria donors and terminal serum creatinine previous to donation are the main risk factors for DGF (142). The presence of DGF is detrimental for graft performance both in short and long-term. Interestingly, DGF is not only associated with a major rate of acute rejection episodes (143–145) but has also shown to be an independent factor for CKaD (143, 144).

Finally, IRI is a bimodal pathogenic way of tissue damage. Though cold storage and donor hypothermia try to minimize cell metabolism during CIT, some cells like renal tubular epithelial cells remain active in a state of hypoxia. In response to hypoxia, their mitochondria increase the production of reactive oxygen species and tend to develop intracellular acidosis. Thus, prolonged CIT will relentlessly lead to cell death and acute tubular necrosis: the ischemia damage. After reperfusion, the microvascular injury caused by ischemia enhances fluid filtration, with leukocyte plugging in capillaries and damaged endothelial cells secreting factors to favor inflammatory mediators and proteolytic enzymes (146). The global outcome of this ischemia-reperfusion damage is a harmful environment that through DAMPS and PAMPS enhances both innate and cellular immunity (147).

Thus, CIT is associated with a decrease in long term graft survival, but its impact is explained for the risk of developing an AR and DGF. Meanwhile, DGF is independently associated with both decreased long term graft survival and increased AR risk. IRI is a pathogenic phenomenon intimately related long CIT and explains DGF, chronic lesions and rejection risk (148).

Currently, no pharmacological intervention has proved to mitigate DGF neither in terms of duration nor its consequences. In clinical practice some strategies are used based on theoretical rationales without evidence. CNI delayed introduction to avoid toxicity and complement activation is one of these strategies although current evidence does not show significant differences (149). Combined to CNI delayed introduction, the use of Anti-Thymocyte Globulins (ATG) has been used to prevent AR and mitigate damage due to IRI. Even though ATG induction seems to have a better security profile in immunological high-risk patients, it is not clear that this grants any advantages in low-risk recipients compared with Basiliximab, not even in terms of reducing DGF (150). The results of PREDICT-DGF trial (NCT02056938, EudraCT #2014-000332-42) will provide more light about if ATG reduce DGF in comparison with Basiliximab in a selected group of patients at a high risk of developing DGF (151). The role of complement on IRI damage has also been tested as a therapeutic target. The use of C1 esterase inhibitor (C1INH) has shown good results in phase I/II studies, especially in those patients with more risk of DGF (152). Novel therapeutic options are also been tested like siRNA, mesenchymal stem cells or thrombin-targeted per-fluorocarbon nanoparticles (PFC-NP) (153, 154). However, all these strategies are far from being used in clinical practice.

Instead, the only strategy that has been proven effective to reduce CIT related DGF is the use of machine perfusion technology during kidney storage. Specifically, Tingle SJ et al. (155) have recently published a meta-analysis on this topic, showing that in deceased donors storage in hypotermic machine perfusion the incidence of DGF was reduced by 23% in comparison with static cold storage solution.

## The Reparation Mechanisms

As reviewed, lots of efforts are currently being put into understanding the whole spectrum of kidney graft damages better. On each field, small strides are being made but with a global vision, CKaD is still worrying. A common limit for all therapeutic strategies is the presence of chronic lesions. These lesions, histologically characterized by fibrosis and extracellular matrix (ECM) deposition, are considered scars which can no longer be repaired. Yet, from another point of view, scars are the result of a wound healing mechanism designed to repair or regenerate kidney damage.

**Figure 1:**
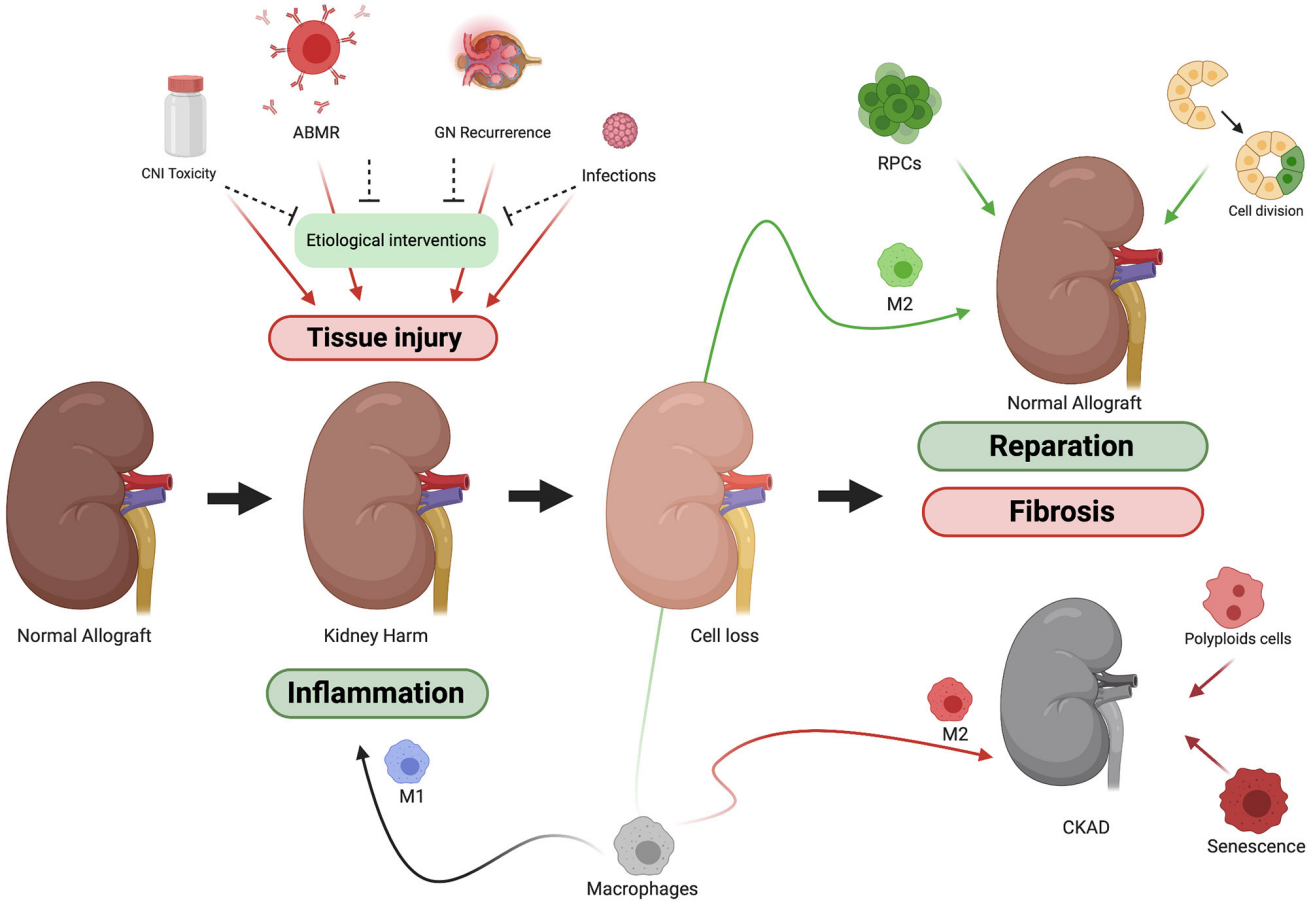
Schematic representation of kidney allograft injury and reparation process. Kidney allograft is exposed to many insults. Although there are mechanisms to protect the graft from each of them, tissue damage is often present at the end. Once the tissue is injured the kidney's owns mechanisms of reparation come into action. To stop the insult, M1 macrophages among others will trigger and inflammatory response. After that, the damage caused (cell loss) must be compensated. Factors like the type and duration of the damage or the age of the donor will determine whether the reparation process tends more toward reparation or scarring (fibrosis).

Schematically, when damage of any etiology happens there is a loss of mass of functioning cells and consequently an attrition in organ function. Therefore, the healing process has to (1) stop the damage, (2) refill the gaps left by cell loss, and (3) compensate the loss of organ function (Figure 1). Probably, the magnitude and duration of the damage, the age of the organism and the dialogue among the effectors of kidney regeneration will determine the balance between healing and regeneration processes. These are the main regeneration mechanisms for kidney regeneration (Table 3).

**Table 3:** Kidney regeneration mechanisms.

**Reparation Mechanism**	**Potential therapeutic target**	**Current limitations**
Machophages	Cell therapy	M1-M2 phenotype plasticity
		Cell obtention
Renal progenitor Cells	Podocite loss biomarker	Pre-clinical phase
	To control crescent formation	
Tubular regeneration	Senolytic stretagies	Pre-clinical phase
	“AKI to CKD” minimization	
Stem cells	MSC-based therapy	Legal/Administration restrictions
		Low-grade evidence

### Macrophages

One of the clearest examples of why the reparations mechanisms are doubled-edged swords is the study of macrophages in CKaD. There is a considerable body of evidence that both circulating and, mainly resident kidney macrophages play a crucial role in kidney inflammation and healing. These hematopoietic cells derived from the yolk sac seem to remain in the kidney during embryogenesis forming niches and they are activated when the harm occurs (156).

After injury, the presence of pathogen-associated molecular patterns (PAMPs) and damage-associated molecular patterns (DAMPs) is recognized via Toll-like receptors (TLR) or patterns recognition-receptors (PRR) and activate a subpopulation of macrophages (M1 or activated macrophage). These activated M1 macrophages are proinflammatory cells capable of secreting pro-inflammatory cytokines (IL-6, IL-1, and TNF-α), superoxide anions and oxygen and nitrogen radicals (157). The amplification of the inflammation cascade contributes to fight against the cause of the injury but as a side effect it does also kill host cells increasing tissue damage. These cells will also contribute to kidney fibrosis through secretion of MMP-9, which increases tubular cell ECM transition via the β-catenin pathway (158). In animal models, the depletion of M1 macrophages ameliorated kidney injury (159). These results were also seen in rat models of acute rejection (160).

In addition, there are other populations of macrophages with distinct function in the reparation process. The alternatively activated macrophages (AAM or M2) usually appear later and have an anti-inflammatory function. Up to three types of M2 macrophages can be differentiated: M2a, stimulated by IL-4 and/or IL-3, are capable of secreting ECM components and therefore they also participate in wound healing and tissue remodeling. M2b have an immunoregulatory profile, inducing IL-10 secretion, upregulating antigen presentation through MHC II and downregulating IL-12, IL-6 and TNF. Finally, M2c macrophages are induced by IL-10, TGF-ß and glucocorticoids and produce anti-inflammatory cytokines (161). In a model with depletion of M2 macrophages, a reduction in tubular cell proliferation and repair is observed (159). Altogether, the macrophage system seems to contribute to kidney regeneration in two differentiated phases. First, immediately after kidney injury M1 population contributes to fight the cause of the aggression through inflammation. Second, once the aggression is neutralized, M1 and M2a contribute to restore the damaged tissue by the production of ECM while M2b and M2c populations reduce inflammation to restore kidney homeostasis.

However, an imbalance in this system can contribute to aggravate kidney damage both in animals and humans (159). For example, macrophage depletion is a useful tool to reduce kidney damage in *in vitro* models, yet a selective depletion to manipulate the M1/M2 ratio has different effects. Moreover, the activation of M2 macrophages that usually results in a reduction of the damage can be the main effector of kidney fibrosis when chronic or constitutive damage occurs (162). Interestingly, Wang et al. demonstrated that after kidney rejection, fibrosis is associated with a constitutive activation of macrophages (mostly M2) especially in chronic active forms. In addition, they identified as etiologic factors of fibrosis not only the production of ECM components but also a macrophage to myofibroblasts transition (163).

Cell therapy using the macrophage system has been attempted in animal models to control kidney fibrosis. The phenotype plasticity of this cell type is one of the main limitations. Cao et al. (164) failed to protect kidney function after infusion of bone narrow derived M2 macrophages, mainly due to phenotypic changes of the infused cells. Spleen macrophages, on the contrary, seem to be more stable and have demonstrated a beneficial effect in model of Adriamycin induced nephropathy.

### Renal Progenitor Cells (RPCs)

In 2006, a population of progenitor cells surrounding the Bowman's capsule was identified. These cells, which are characterized by the co-expression of CD133 and CD24 markers, display a multipotent capacity of evolving into kidney specific cells terminally differentiated (podocytes and tubular cells) (165). Through differentiation, these RPCs exhibit the capacity to ameliorate acute kidney damage (166). At the same time, under certain conditions, as a sustained injury, these cells could also contribute to crescent formation (167) or glomerulosclerosis (16, 168). Sicking et al. (169) observed how after podocyte damage caused by doxycycline, RPCs from Bowman's capsule tended to leave their position to replace podocytes. After, the remaining RPCs formed cellular extensions to cover the denuded Bowman's capsule surface (expressing *de novo* CD44). Throughout the observation period, the induced proliferation of RPCs persisted, resulting in the formation of typical cellular crescents with periglomerular infiltrate. Thus, it can be hypothesized that RPCs act as a physiological renal regenerative mechanism that when overcome tend to scar in order to prevent further damage. Importantly, urinary detection of RPCs is feasible and has been already used to perform functional and genotypic studies without the need of invasive procedures (170, 171).

Recently, Manonelles et al. (172) published the first and only experience of RPCs isolation in kidney transplant recipients. In this study, a cohort of stable kidney transplant recipients with 6 months protocol biopsy was divided into two groups depending on the presence or absence of urinary RPC. A total cohort of 66 patients were then followed for 2 years. Interestingly, at the beginning of the study both groups were identical considering clinical variables, alloimmune response, renal function, albuminuria and graft pathology. However, uRPC+ group showed increased podocyturia and a higher rate of proliferating RPCs along the Bowman's capsule, suggesting that RPCs were proliferating to compensate a podocyte loss. Consequently, 2 years follow up evidenced poorer outcomes in the uRPC+ group with worse renal function, increased albuminuria, wider mesangial expansion and more severe interstitial fibrosis. If these results are confirmed, the detection of urinary RPCs could act as a marker of current injury much before clinical, immunological or histological damage is detected. Whether a therapeutic intervention at this time could prevent function graft attrition must be proved in further studies.

### The Tubular Regeneration: Dedifferentiation and Polyploidization

Tubular epithelium is the kidney structure that most commonly suffers damage due to its high metabolic activity and decreased blood supply. Tubular regeneration model has great efficiency and is able to repair after an injury with no or little consequences. One of the important characteristics in this model is that tubular epithelial cells (TECs) are simpler than other kidney cells like podocytes. This feature allows TECs to easily dedifferentiate after an injury in order to do mitosis and repopulate the epithelia (173). This model for regeneration through dedifferentiation has been classically proposed for tubular regeneration but some objections have been done recently.

Lazzeri et al. suggested that the regeneration capacity of the tubule through dedifferentiation had largely been overestimated. Instead, they proposed a model of regeneration highly preserved in other human organs (174). This model is based on two main effectors: first, niches of progenitor cells can be found near the remaining tissue in a non-differentiated state. Second, after injury the remaining differentiated cells increase its content in DNA without undergoing mitosis through a specific cell cycle called endocycle. Thus, these cells can improve their performance reducing the loss of function of the organ while complete regeneration occurs. The same group has already demonstrated the presence of niches of cells that express progenitor markers next to the tubule and also the existence of endocycling cells, in both animals and humans (175).

In this model, the injury would cause a cell loss that implies decrease of organ of function of the organ. After that, progenitor cells would be mobilized from their niches and would finalize their differentiation process to repopulate the epithelia. In the meantime, to preserve organ function (or even survival) the remaining tubular cells would enter endocycle in order to duplicate its DNA content and keep the function of the organ. These cells though, will not be able to perform a normal cell cycle ever again; so, they are doomed to become a hyperfunctioning cell with a hypersecretory state of profibrotic mediators driving fibrogenesis, that is, cell senescence (176). These aberrant profibrotic cells would be responsible for fibrosis after AKI explaining, at least partially, the AKI-to-CKD transition. The balance between progenitors' repopulation and endocycling cells would then be crucial in a complete regeneration of the organ without later damage. Consistently, it has been observed that, the amount of progenitor cells in mammals decrease with aging and endocycling cells take a more important role in reparation processes, which would lead to greater fibrosis (177).

### Stem Cells

To involve the kidney's own repair processes as a therapeutic tool is a strategy that has been already tested with promising results through the infusion of stem-cells. In general, based on their therapeutic potential, mesenchymal stem cells (MSCs) from bone marrow or other tissues are considered one the most powerful tools for treating several human diseases. MSC action is based not only on the capacity to differentiate into terminal renal cells but they have also been associated with the release of pro-mitotic, anti-apoptotic, anti-inflammatory and immunomodulatory soluble factors as well as to the mitigation of metabolomic and oxidative stress imbalance (178). A number of clinical trials have been designed to evaluate the safety and efficacy of MSC-based therapy and some good results have been observed in acute kidney injury trials (179). Very recently, the TRITON study (180) has used the infusion of autologous MSCs in 29 kidney transplant recipients to withdraw CNI. After 24 weeks of follow up, no differences were observed in graft function, acute rejection, graft loss, major adverse events or in kidney fibrosis. In a *post-hoc* analysis of this study, a longer follow up (5-year) was performed observing a more preserved renal function in the MSC group. Although there are already many limitations and restrictions to cell therapy, this study shows the feasibility of these treatments and which could be a cornerstone in future kidney transplantation therapeutic regimens.

## Conclusion

Long-term graft survival is a major concern in the transplant community due to its clinical impact. Until now, lots of efforts have been put into identifying and precisely mitigate the impact of every potential graft damage. Consequently, advances in the treatment of ABMR are expected to report greater outcomes than BK virus or pyelonephritis prevention. However, from a pragmatic point of view, all the aforementioned harmful situations will be present in every kidney graft contributing to the final outcome: Chronic Kidney Allograft Disease. CKaD needs to be addressed by a holistic strategy. A therapeutic approach that considers to abrogate the mechanisms of graft injury and to improve the intrinsic mechanisms of kidney repair could have a transversal impact and lead to a significant improvement in CKaD. Further studies are needed to address this issue in the coming years.

## Author Contributions

SC reviewed the literature and prepared the manuscript. AM, MT, and AS reviewed the manuscript. JC supervised and reviewed the manuscript. All authors approve this manuscript for publication.

## Conflict of Interest

The authors declare that the research was conducted in the absence of any commercial or financial relationships that could be construed as a potential conflict of interest.
